# Subtypes and service utilization among opioid use disorder patients at a community health center: findings from a medically underserved urban area of the Northeastern United States

**DOI:** 10.1186/s13722-025-00564-z

**Published:** 2025-05-08

**Authors:** Orrin D. Ware, Jamey J. Lister, Sarah E. Cooper, Andrew H. Kim, Holly H. Lister, N. Andrew Peterson, Stephen Fioravanti, Kristen Gilmore Powell, Stephanie C. Marcello, Bethany Joseph

**Affiliations:** 1 School of Social Work, University of North Carolina at Chapel Hill, Chapel Hill, NC USA; 2 School of Social Work, Rutgers, The State University of New Jersey, New Brunswick, NJ USA; 3 Center for Integrated Care, University Behavioral Health Care, Rutgers Health, Piscataway, NJ USA; 4 Trinitas Regional Medical Center, Robert Wood Johnson Barnabas Health, Elizabeth, NJ USA

**Keywords:** Co-Occurring, Behavioral health, Dual diagnosis, Healthcare coverage, Opioid use disorder, Service, Substance use, Treatment

## Abstract

**Background:**

Opioid use disorder often co-occurs with other mental health and substance use disorders. Identifying clusters of individuals receiving treatment for opioid use disorder based on co-diagnosed conditions, healthcare plans, and service utilization over a seven-year treatment period provides insight into service needs. Objectives included [1] characterizing the sample [2], examining subtypes of the sample using cluster analysis, and [3] identifying differences in Current Procedural Terminology by subtype to examine service utilization among identified clusters.

**Methods:**

This study uses secondary data from the electronic medical records of a community health center in a large urban area in the Northeastern United States from 2015 to 2021. The study sample included *N* = 705 adults who had an opioid use disorder diagnosis as indicated by the community health center’s electronic medical records. Measures include [1] age [2], race and ethnicity [3], sex [4], healthcare plan(s) [5], co-occurring mental health disorder [6], co-occurring substance use disorder [7], co-occurring mental health disorder or substance use disorder, and [8] Current Procedural Terminology codes for behavioral health service utilization. Cluster analysis was used to examine the sample. These clusters were then analyzed for service utilization with a one-way analysis of variance.

**Results:**

The cluster analysis identified six clusters with an average silhouette of 0.5, indicating good clustering. These six clusters were operationalized as [1] Medicare/Medicaid healthcare plan with substance use disorder needs [2], Private pay and charity care healthcare plan with cocaine use disorder needs [3], Medicare/Medicaid and other publicly-funded healthcare plans with mood disorder needs [4], Private healthcare plan with low co-occurring disorder needs [5], Other publicly-funded healthcare plan with cannabis use disorder needs [6], Medicare/Medicaid healthcare plan with mental health disorder needs. Service utilization differed between these clusters with cluster mean differences for psychotherapy sessions (*F* = 8.55, *p* < 0.001), psychiatric sessions (*F* = 22.72, *p* < 0.001), and group therapy sessions (*F* = 10.76, *p* < 0.001).

**Conclusions:**

This study highlights the importance of comprehensive and integrated treatment for substance use disorders and mental health disorders, particularly for those in underserved communities. Healthcare coverage, a socioeconomic factor that impacts access to care, is critical in distinguishing treatment needs and utilization.

**Supplementary Information:**

The online version contains supplementary material available at 10.1186/s13722-025-00564-z.

## Introduction

Impacts of substance use disorders (SUDs) and mental health disorders (MHDs) on the population’s health are pronounced by their co-occurrence. Approximately 10% of adults in the United States (U.S.) have co-occurring SUD and MHD [1], and having one condition is associated with an increased risk of developing another [2–4]. Opioid use disorder (OUD), one of the most prevalent SUDs, is particularly concerning considering opioid-related overdoses; [5–8] 1 in 22 deaths in the U.S. were attributed to opioid toxicity in 2021 [9]. Individuals with OUD are likely to have had a MHD or another SUD in their lifetime [10–13]. Considering the complex clinical needs of individuals with OUD who have co-occurring MHDs and/or SUDs, it is necessary to screen and provide integrated treatment that addresses co-morbidity and/or multimorbidity.

Despite the need for treatment among persons with OUD, people often face barriers to evidence-based services. Financial barriers include not having insurance or being able to self-pay [14, 15]. Medicaid and payment assistance are options intended to decrease financial barriers [5, 16, 17]. The Affordable Care Act was implemented to increase Medicaid eligibility [17, 18] allowing states to adopt Medicaid expansion. The state of New Jersey (NJ), adopted this expansion on January 1, 2014 [18]. Further, NJ provides Charity Care (pay assistance) for persons meeting income and asset requirements [19]. Utilizing flexible payment options such as Charity Care, Medicare, and Medicaid may increase treatment engagement.

Community health centers often provide primary care for local individuals with poorer health statuses and a lower socioeconomic status than the general population [20, 21]. Since most individuals receiving care from health centers have incomes below the federal poverty level, these facilities adjust service costs based on income and size of the family to increase access [20, 22]. As such, community health centers are robust settings to measure treatment characteristics and engagement among individuals with financial barriers. These clinics represent a diverse patient population with high treatment needs and significant barriers to care. It is essential that we not only improve our understanding of the complex needs of individuals with co-occurring disorders, including individuals with OUD, but that we also do so with a lens toward the disparities in care access.

While often used for administrative and reimbursement purposes, data from real-world treatment facilities such as community health centers can provide a snapshot into real-world samples receiving treatment [23, 24]. As individuals with OUD may have co-occurring diagnoses [10–13], and may face barriers to accessing treatment [14, 15], administrative data from community health centers provides an avenue to identify different patient groupings and how those groups utilize services. Although community health centers already provided care to individuals with a lower socioeconomic status [20–22], NJ was an early adopter of Medicaid expansion in 2014, potentially increasing service access [18]. Taken together, this current study sought to identify different patient groups (based on diagnoses and healthcare plans) and service utilization among patients receiving care from a community health center located in an early Medicaid expansion state. This study can identify barriers and facilitators in service utilization based on real-world diagnoses and healthcare plans.

This study examines adults with OUD receiving treatment in NJ at a community health center from 2015 to 2021. Aims included [1] characterizing the sample [2], examining subtypes of the sample using a cluster analysis, and [3] identifying differences in behavioral health service utilization using Current Procedural Terminology (CPT) codes by subtype. Cluster groupings were examined by healthcare plan options, co-occurring MHDs, and co-occurring SUDs during this study period. We focused on the sub-sample of people with OUD diagnoses to focus on an SUD associated with high mortality risks related to overdoses. The specific study years, 2015–2021, provide the opportunity to examine these clusters seven years after Medicaid expansion in NJ.

## Methods

### Data

This study used secondary data from the electronic medical records of a community health center in a large urban area of NJ. Included patients were receiving care in a medically underserved community in one of the most culturally diverse and representative states in the nation. Cases include persons aged 18–65 years old who were screened for substance misuse when they received health services from 2015 to 2021. The dataset, containing 6,780 unique persons identified by medical record numbers (MRNs), was transferred to the research team from the clinical site. MRNs were used to link patient information across multiple visits during the study’s analytic period (2015–2021). To address this study’s aims, we selected only persons in the dataset with an OUD diagnosis, resulting in a sample of *N* = 705 adults. Variables of interest include [1] age [2], race and ethnicity [3], sex [4], healthcare plan(s) [5], co-occurring MHD [6], co-occurring SUD [7], co-occurring MHD or SUD, and [8] CPT code for behavioral health service utilization.

### Measures

*Age*. This continuous variable identified a person’s age in years on their first admission.

*Race and Ethnicity*. The race and ethnicity of an individual in the dataset was identified by five categories. Initial race and ethnic data included open-ended self-identified racial and ethnic identities. Responses were collapsed to conform with the U.S. Census Bureau racial and ethnic survey categories. These categories are [1] Black or African American [2], Hispanic of any Race [3], White [4], Another Race or Ethnicity, and [5] Unknown Race or Ethnicity. The “Another Race or Ethnicity” variable included categories that were endorsed by ≤ 5 cases, including “American Indian and Alaska Native”, “Asian”, “Native Hawaiian and other Pacific Islander”, “Two or More Races”, and “Other”. This variable was captured during an individual’s first admission. Hereafter, Black or African American and White race imply Non-Hispanic ethnicity.

*Sex*. This variable describes a person’s biological sex as female or male, captured during the first admission.

*Healthcare Plan*. Healthcare plan describes a group of five non-mutually exclusive variables. These variables initially included several specific factors (e.g., Aetna Better Health, United Healthcare Medicare, Probation, etc.), which were then collapsed into broader categories for analysis. These binary (Yes/No) variables are [1] Charity Care (i.e. Payment Assistance) [2], Medicare/Medicaid [3], Private Insurance [4], Private Pay, and [5] Other Funding (e.g., Drug Court). Each variable describes whether any of these five healthcare plans was used for clinical services received during the analytic period.

*Co-occurring Mental Health Disorder (MHD)*. This variable described whether individuals were diagnosed with a MHD during the study period. Specifically, this study focused on five MHD groupings outlined in the Diagnostic Statistical Manual 5 Text Revision (DSM-5 TR) [25] and how national mental health treatment data are presented [26], including [1] anxiety disorders [2], bipolar disorders [3], depressive disorders [4], schizophrenia spectrum disorders, and [5] trauma- and stressor-related disorders. Diagnostic data originally involved a wide range of ICD F codes (e.g., major depressive disorder; specific ICD codes may be seen in the supplemental document), which we collapsed within umbrella grouping categories (i.e., depressive disorders) and contained specifiers (moderate, severe, etc.) that were not retained when categorizing diagnoses by their grouping. The five MHD groupings in this study are some of the most common among adults. As other MHD diagnoses in the dataset had low levels of endorsement and low rates in the general population, they were omitted.

*Co-occurring Substance Use Disorder (SUD)*. This variable described whether individuals in the dataset were diagnosed with another SUD (not OUD since OUD diagnosis was selection criteria) during the study period. Diagnoses included [1] alcohol use disorder [2], cannabis use disorder [3], cocaine use disorder, and [4] other substance use disorder. Similar to MHDs, diagnosis data involved a wide range of ICD codes which were collapsed within umbrella grouping categories and originally contained specifiers which were not retained when categorizing diagnoses by their grouping in the DSM-5 TR.

*Co-occurring MHD or SUD*. This variable combined the “Co-occurring Mental Health Disorder” and “Co-occurring Substance Use Disorder” variables into a categorical variable with four values: [1] No Co-occurring MHD or SUD [2], Co-occurring SUD but no MHD [3], Co-occurring MHD but no SUD [4], Co-occurring MHD and SUD.

*CPT Codes for Behavioral Health Service Utilization.* Service utilization codes for three behavioral health service type domains were included in this analysis: [1] individual psychotherapy sessions [2], evaluation and treatment sessions with psychiatrists, and [3] group therapy sessions. Each service variable was collapsed across all years to create a total number of sessions for each of the three service types.

### Ethical review

Study procedures were approved by the Rutgers University Institutional Review Board, Protocol Number 2021001768 (Principal Investigator: JJL).

### Analysis

IBM SPSS Statistics Version 29 was used [27]. Univariable statistics described the sample. With the maximum memory allocation set to 64 megabytes, a two-step cluster analysis using Bayesian Information Criteria, log-likelihood distance measure, and a maximum of fifteen clusters as the default maximum number of clusters examined groupings. Two-step cluster analysis uses an algorithm to examine different groupings based on provided variables that are independent of one another [28]. The average silhouette score, which measures cluster quality, was examined. The initial cluster analysis included: age, race and ethnicity, sex, healthcare plan, co-occurring MHD, co-occurring SUD, and co-occurring MHD and SUD. The initial analysis indicated poor clustering (average silhouette = 0.3). A final cluster analysis was conducted, which included variables that could be endorsed multiple times throughout the analytic period: healthcare plan and co-occurring MHD or SUD. After conducting the cluster analysis, a one-way analysis of variance (ANOVA) was conducted to determine if there were significant differences in age between the different clusters, with Bonferroni for post hoc analysis. Chi-square was used to examine differences in groups by categorical variables. Adjusted standardized residuals (ASRs) were then computed to examine post hoc differences between clusters using a threshold of ≤ -2 and ≥ 2, in line with Haberman’s rule of thumb and prior literature [29, 30]. ANOVAs were conducted to examine group differences in CPT codes for behavioral health service utilization among the clusters, with Bonferroni post-hoc tests [31]. 

## Results

### Sample description

As seen in Table 1, 40.0% of the sample were Black or African American, and 70.6% were male. Endorsed non-OUD SUDs were cannabis use disorder (31.8%), cocaine use disorder (31.6%), and alcohol use disorder (28.4%). Percentages of the most prevalent MHD diagnoses included depressive disorders (19.4%) and bipolar disorders (12.8%).

**Table 1:** Descriptive characteristics of the analytic sample and clusters

	Entire Analytic Sample *N* = 705 (*n*, %)	Cluster 1 *n* = 82 (*n*, %)	Cluster 2 *n* = 117 (*n*, %)	Cluster 3 *n* = 120 (*n*, %)	Cluster 4 *n* = 121 (*n*, %)	Cluster 5 *n* = 135 (*n*, %)	Cluster 6 *n* = 130 (*n*, %)
Age, Mean (SD)^1^	44.1 (12.1)	43.7 (12.0)	41.6 (11.7)^#^	43.6 (11.4)	44.0 (13.2)	43.8 (11.3)	47.4 (12.5)^#^
Race and Ethnicity^1^							
Black or African American	282 (40.0%)	33 (40.2%)	39 (33.3%)	46 (38.3%)	46 (38.0%)	61 (45.2%)	57 (43.8%)
Hispanic of any Race	117 (16.6%)	14 (17.1%)	19 (16.2%)	24 (20.0%)	29 (24.0%)^b^	11 (8.1%)^a^	20 (15.4%)
White	263 (37.3%)	29 (35.4%)	52 (44.4%)	42 (35.0%)	37 (30.6%)	54 (40.0%)	49 (37.7%)
Another Race or Ethnicity	12 (1.7%)	2 (2.4%)	1 (0.9%)	3 (2.5%)	3 (2.5%)	2 (1.5%)	1 (0.8%)
Unknown Race or Ethnicity	31 (4.4%)	4 (4.9%)	6 (5.1%)	5 (4.2%)	6 (5.0%)	7 (5.2%)	3 (2.3%)
Sex^1^							
Female	207 (29.4%)	23 (28.0%)	32 (27.4%)	41 (34.2%)	41 (33.9%)	24 (17.8%)^a^	46 (35.4%)
Male	498 (70.6%)	59 (72.0%)	85 (72.6%)	79 (65.8%)	80 (66.1%)	111 (82.2%)^b^	84 (64.6%)
Healthcare Plan							
Charity Care	72 (10.2%)	0 (0.0%)^a^	68 (58.1%)^b^	0 (0.0%)^a^	4 (3.3%)^a^	0 (0.0%)^a^	0 (0.0%)^a^
Medicare/Medicaid	527 (74.8%)	82 (100.0%)^b^	95 (81.2%)	120 (100.0%)^b^	100 (82.6%)^b^	0 (0.0%)^a^	130 (100.0%)^b^
Private Insurance	158 (22.4%)	0 (0.0%)^a^	11 (9.4%)^a^	25 (20.8%)	121 (100.0%)^b^	1 (0.7%)^a^	0 (0.0%)^a^
Private Pay	147 (20.9%)	0 (0.0%)^a^	108 (92.3%)^b^	0 (0.0%)^a^	24 (19.8%)	15 (11.1%)^a^	0 (0.0%)^a^
Other Funding	323 (45.8%)	0 (0.0%)^a^	41 (35.0%)^a^	120 (100.0%)^b^	27 (22.3%)^a^	135 (100.0%)^b^	0 (0.0%)^a^
Co-Diagnosed SUD Dx							
Alcohol Use Disorder Dx	200 (28.4%)	33 (40.2%)^b^	30 (25.6%)	40 (33.3%)	31 (25.6%)	39 (28.9%)	27 (20.8%)^a^
Cannabis Use Disorder Dx	224 (31.8%)	33 (40.2%)	37 (31.6%)	44 (36.7%)	36 (29.8%)	54 (40.0%)^b^	20 (15.4%)^a^
Cocaine Use Disorder Dx	223 (31.6%)	35 (42.7%)^b^	48 (41.0%)^b^	39 (32.5%)	42 (34.7%)	26 (19.3%)^a^	33 (25.4%)^a^
Other Substance Use Disorder Dx	112 (15.9%)	19 (23.2%)	19 (16.2%)	18 (15.0%)	18 (14.9%)	23 (17.0%)	15 (11.5%)
Co-Diagnosed MHD Dx							
Anxiety Disorder Dx	76 (10.8%)	0 (0.0%)^a^	13 (11.1%)	18 (15.0%)	10 (8.3%)	9 (6.7%)	26 (20.0%)^b^
Bipolar Disorder Dx	90 (12.8%)	0 (0.0%)^a^	17 (14.5%)	26 (21.7%)^b^	12 (9.9%)	6 (4.4%)^a^	29 (22.3%)^b^
Depressive Disorder Dx	137 (19.4%)	0 (0.0%)^a^	16 (13.7%)	38 (31.7%)^b^	24 (19.8%)	13 (9.6%)^a^	46 (35.4%)^b^
Schizophrenia Spectrum Disorder Dx	30 (4.3%)	0 (0.0%)^a^	6 (5.1%)	3 (2.5%)	7 (5.8%)	1 (0.7%)^a^	13 (10.0%)^b^
Trauma- and Stressor-Related Disorder Dx	50 (7.1%)	0 (0.0%)^a^	8 (6.8%)	9 (7.5%)	10 (8.3%)	5 (3.7%)	18 (13.8%)^b^
Co-Diagnosed MHD or SUD							
No Co-Diagnosed MHD or SUD	123 (17.4%)	0 (0.0%)^a^	20 (17.1%)	21 (17.5%)	12 (9.9%)^a^	34 (25.2%)^b^	36 (27.7%)^b^
Co-Diagnosed SUD but no MH	308 (43.7%)	82 (100.0%)^b^	54 (46.2%)	33 (27.5%)^a^	62 (51.2%)	77 (57.0%)^b^	0 (0.0%)^a^
Co-Diagnosed MHD but no SUD	78 (11.1%)	0 (0.0%)^a^	10 (8.5%)	13 (10.8%)	21 (17.4%)^b^	6 (4.4%)^a^	28 (21.5%)^b^
Co-Diagnosed MHD and SUD	196 (27.8%)	0 (0.0%)^a^	33 (28.2%)	53 (44.2%)^b^	26 (21.5%)	18 (13.3%)^a^	66 (50.8%)^b^

Dx = Diagnosis

MHD = Mental Health Disorder

SUD = Substance Use Disorder

^1^Sociodemographic variables are included in this table for descriptive purposes and were not included in the cluster analysis

^a^Adjusted Standardized Residual (ASR) = ≤ -2

^b^Adjusted Standardized Residual (ASR) = ≥ 2

^#^Significant group differences in Bonferroni posthoc comparisons

### Identified clusters

**Fig. 1:**
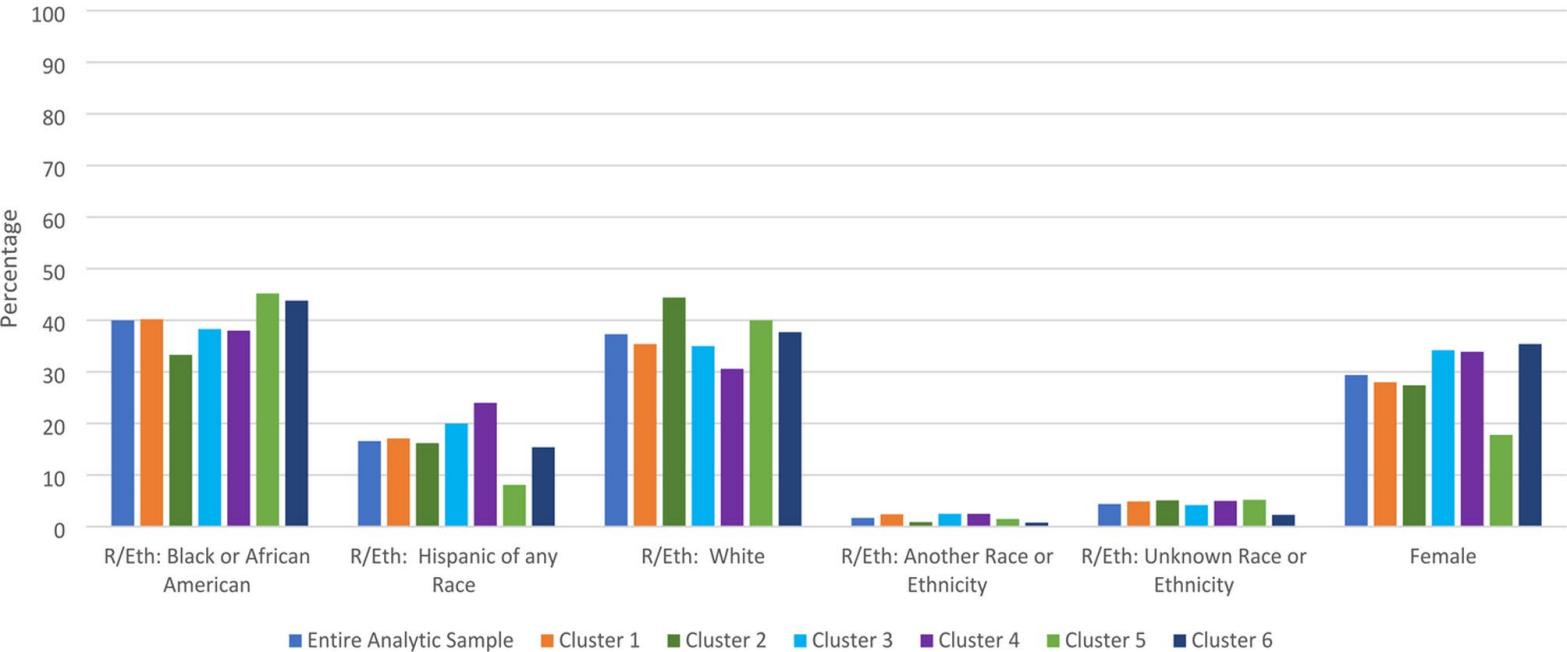
Demographic percentages within cluster

Six clusters were identified, with an average silhouette of 0.5, indicating good clustering. Table 1 and the Figures provide cluster characteristics (Fig 1).

### Cluster 1: “Medicare/Medicaid healthcare plan with SUD needs”

Cluster 1 is a homogeneous group of patients regarding payment and co-morbidity. All of Cluster 1 used Medicare/Medicaid as their healthcare plan (100.0%, ASR = 5.6; *X*^*2*^ = 518.396, *p* < 0.001). All patients had a co-morbid SUD diagnosis but no MHD diagnosis status (100.0%, ASR = 10.9; *X*^*2*^ = 264.708, *p* < 0.001). Specifically, Cluster 1 had a large proportion of cocaine use disorder (42.7%, ASR = 2.3; *X*^*2*^ = 21.879, *p* < 0.001) and alcohol use disorder (40.2%, ASR = 2.5; *X*^*2*^ = 11.737, *p* = 0.039). While 40.2% of Cluster 1 had cannabis use disorder, this did not differ from other clusters.

### Cluster 2: “Private pay and charity care healthcare plan with cocaine use disorder needs”

Cluster 2 had a mixture of healthcare plans, though it had the largest proportion (and unique from other clusters) of private pay (92.3%, ASR = 20.8; *X*^*2*^ = 457.289, *p* < 0.001) and Charity Care (58.1%, ASR = 18.7; *X*^*2*^ = 352.249, *p* < 0.001). Cluster 2 had a significantly larger proportion of cocaine use disorder (41.0%, ASR = 2.4; *X*^*2*^ = 21.879, *p* < 0.001) compared to other clusters.

### Cluster 3: “Medicare/Medicaid and other publicly-funded healthcare plans with mood disorder needs”

Cluster 3 is a homogenous group regarding healthcare plans as Medicare/Medicaid (100.0%, ASR = 7.0; *X*^*2*^ = 518.396, *p* < 0.001) and the other healthcare plan category (100.0%, ASR = 13.1; *X*^*2*^ = 513.226, *p* < 0.001) were uniformly endorsed. The only other healthcare plan identified in Cluster 3 was private insurance (20.8%), though the endorsement wasn’t different from other clusters. Cluster 3 had the largest proportion of patients with both a co-occurring MHD and SUD, with nearly half the cluster co-diagnosed (44.2%, ASR = 13.1; *X*^*2*^ = 264.708, *p* < 0.001). Mood disorders were endorsed at higher proportions than other clusters, specifically depressive disorder (31.7%, ASR = 3.7; *X*^*2*^ = 63.155, *p* < 0.001) and/or a bipolar disorder (21.7%, ASR = 3.2; *X*^*2*^ = 40.768, *p* < 0.001). While other SUD and MHD diagnoses were reported, rates did not differ from other clusters.

### Cluster 4: “Private healthcare plan with low co-occurring disorder needs”

All patients in Cluster 4 utilized private insurance (100.0%, ASR = 22.5; *X*^*2*^ = 528.160, *p* < 0.001), which was the most of any cluster. In terms of diagnoses, Cluster 4 had the second largest proportion of those with a MHD but no co-morbid SUD (17.4%, ASR = 2.4; *X*^*2*^ = 264.708, *p* < 0.001). However, no specific MHD had a notably larger proportion (ASRs ranged from − 1.0 to 0.9).

### Cluster 5: “Other publicly-funded healthcare plan with cannabis use disorder needs”

All of Cluster 5 utilized the other publicly-funded plan (100.0%, ASR = 14.1; *X*^*2*^ = 513.226, *p* < 0.001). Only private pay (11.1%) and private insurance (0.7%) were also utilized by Cluster 5. In terms of diagnosis status, slightly more than half the cluster had a co-morbid SUD but no MHD (57.0%, ASR = 3.5; *X*^*2*^ = 264.708, *p* < 0.001). Cluster 5 also had the second greatest proportion of patients with cannabis use disorder (40.0%, ASR = 2.3) (*X*^*2*^ = 24.591, *p* < 0.001), just behind Cluster 1 at 40.2%. However, Cluster 5 was the only cluster to differ from other clusters in ASR tests.

### Cluster 6: “Medicare/Medicaid healthcare plan with MHD needs”

All of Cluster 6 utilized Medicare/Medicaid (100.0%, ASR = 7.3; *X*^*2*^ = 518.396, *p* < 0.001). No other healthcare plan was utilized by any patient in Cluster 6. Nearly three-quarters of the patients in Cluster 6 had a MHD (MHD only = 21.5%, ASR = 14.2; both MHD and SUD = 50.8%, ASR = 13.1; *X*^*2*^ = 264.708, *p* < 0.001). For all specific MHD diagnoses, Cluster 6 had the greatest proportions of depressive disorder (35.4%; ASR = 5.1; *X*^*2*^ = 63.155, *p* < 0.001), bipolar disorder (22.3%; ASR = 3.6; *X*^*2*^ = 40.768, *p* < 0.001), anxiety disorder (20.0%; ASR = 3.8; *X*^*2*^ = 26.804, *p* < 0.001), schizophrenia spectrum disorder (10.0%; ASR = 3.1; *X*^*2*^ = 20.089, *p* = 0.001), and trauma- and stressor-related disorder (13.8%; ASR = 3.3; *X*^*2*^ = 17.906, *p* = 0.003).

### Notable demographic differences between clusters

**Fig. 2:**
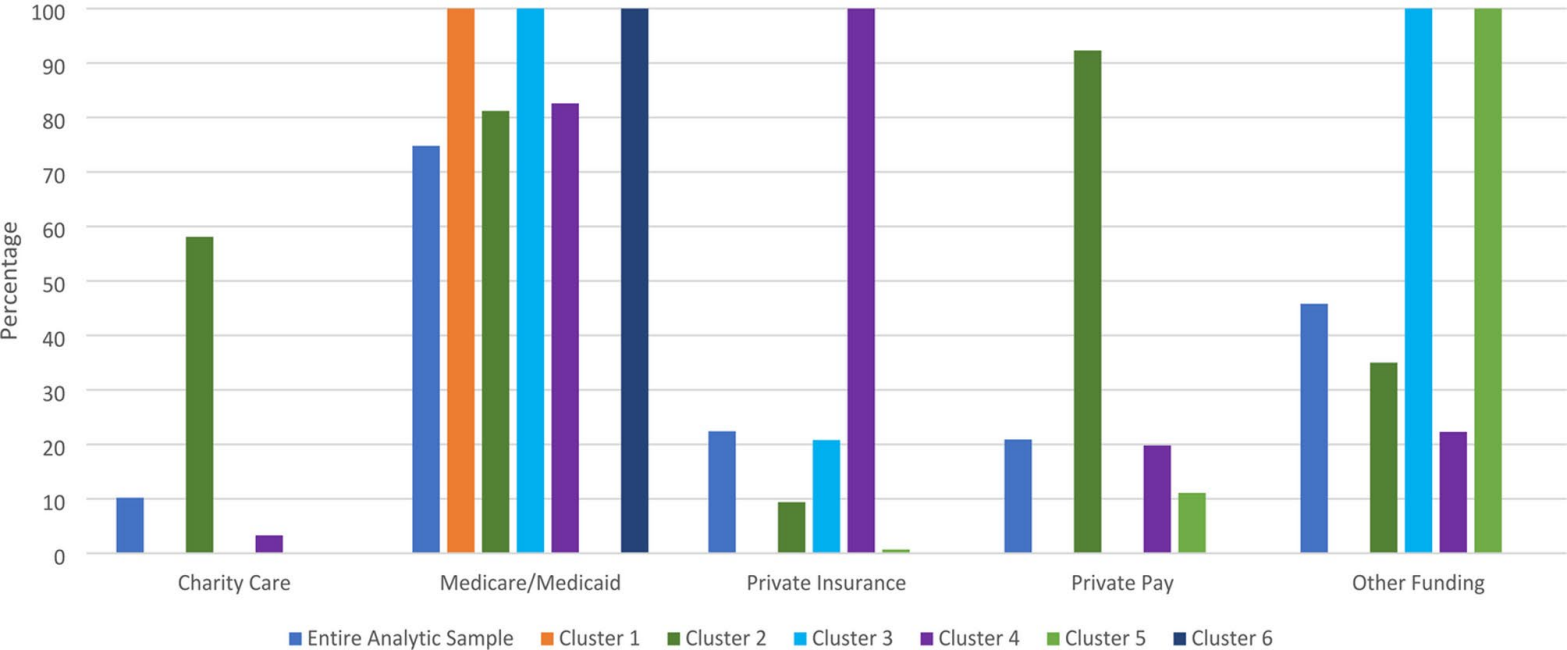
Healthcare plan percentages within cluster

Cluster 2 is notable as being younger (compared to Cluster 6) with a mean age of 41.6 years old (*p* = 0.002, 95% Confidence Interval [-10.2, -1.4]). Cluster 4 had the largest proportion of patients that identified as Hispanic of any race (24.0%, ASR = 2.4), which was meaningfully different from other clusters in post-hoc tests, despite a non-significant group difference (X^2^ = 21.324, *p* = 0.378). Cluster 5 is notable for being comprised of 82.2% men (ASR = 3.3; *X*^*2*^ = 13.833, *p* = 0.017), which is the largest proportion of all the clusters. Cluster 6 had older patients compared to Cluster 2 with a mean age of 47.4 years old (*p* = 0.002, 95% Confidence Interval [-10.2, -1.4]) (Fig 2).

### Service utilization differences

**Fig. 3:**
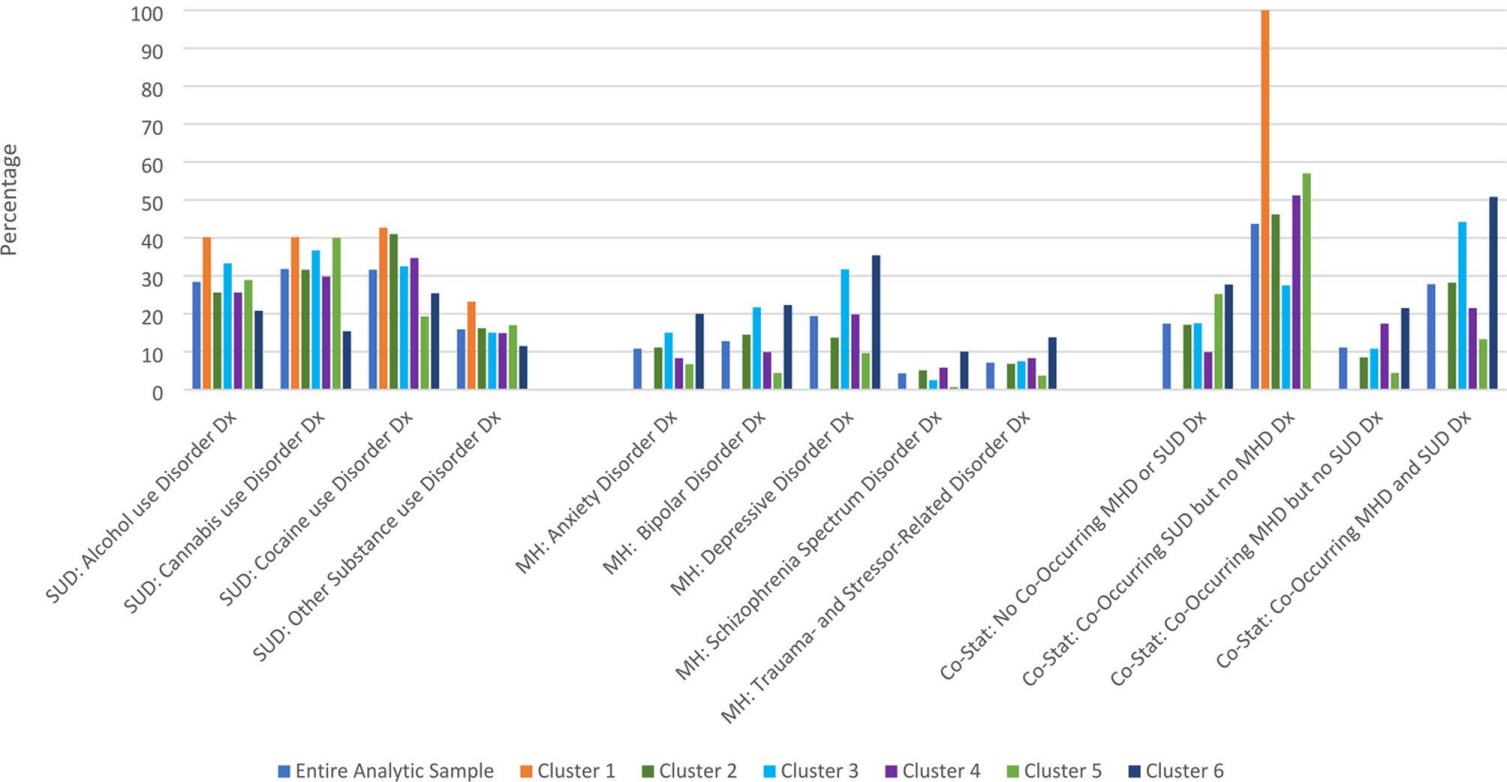
Diagnoses percentages within cluster. *Note*. Co-stat refers to co-occurring diagnosis status

A One-Way ANOVA identified significant cluster mean differences in CPT codes for behavioral health service utilization for psychotherapy (*F* = 8.55, *p* < 0.001), psychiatric (*F* = 22.72, *p* < 0.001), and group therapy sessions (*F* = 10.76, *p* < 0.001; Table 2). When examining specific cluster-to-cluster differences in post-hoc tests (see Table 3), Cluster 3 (“Medicare/Medicaid and other publicly-funded healthcare plans with mood disorder needs”) demonstrated greater utilization for each measure in all but 2 of the 15 post-hoc tests. Cluster 5 (“Other publicly-funded healthcare plan with cannabis use disorder needs”) had differential utilization compared to other clusters in the majority (9 of 15) of post-hoc tests. However, the direction was different by measure, with Cluster 5 demonstrating higher utilization than all but one cluster for psychotherapy sessions, while demonstrating lower utilization with all but one cluster for psychiatric and evaluation sessions. Aside from the differences with Clusters 3 and 5, the only remaining post-hoc differences for Cluster 1 (“Medicare/Medicaid healthcare plan with SUD needs”), Cluster 2 (“Private pay and Charity Care healthcare plan with cocaine use disorder needs”), Cluster 4 (“Private healthcare plan with low co-occurring disorder needs”), and Cluster 6 (“Medicare/Medicaid healthcare plan with MHD needs”) with one another was for psychiatric and evaluation sessions (Cluster 2 had higher utilization than Clusters 1 and 6). While we focus our directional interpretation on a greater number of sessions, it is noteworthy that Clusters 1 and 6, both characterized in part by Medicaid/Medicare healthcare plans and behavioral health needs, demonstrated lower utilization means compared to the full sample for all three measures. Contrastingly, Cluster 3, similarly characterized by Medicare/Medicaid and behavioral health needs, alongside *other publicly-funded healthcare plans*, had greater utilization (Fig 3).

**Table 2:** Average number of behavioral health sessions by cluster

Clusters		Number of Psychotherapy Sessions	Number of Psychiatric Eval & Tx Sessions	Number of Group Therapy Sessions
Entire Analytic Sample	*N* = 705	5.63 (7.25)	1.88 (1.12)	34.02 (39.84)
Cluster 1: “Medicare/Medicaid healthcare plan with SUD needs”	*n* = 82	3.80 (5.14)	1.55 (0.90)	25.55 (37.34)
Cluster 2: “Private pay and Charity Care healthcare plan with cocaine use disorder needs”	*n* = 117	4.98 (6.08)	2.24 (1.39)	37.00 (38.62)
Cluster 3: “Medicare/Medicaid and other publicly-funded healthcare plans with mood disorder needs”	*n* = 120	7.90 (8.65)	2.52 (1.28)	55.97 (55.47)
Cluster 4: “Private insurance + with low co-occurring needs”	*n* = 121	4.74 (7.51)	1.93 (1.07)	30.18 (40.24)
Cluster 5: “Other publicly-funded healthcare plan with cannabis use disorder needs”	*n* = 135	7.84 (8.14)	1.27 (0.57)	28.84 (21.90)
Cluster 6: “Medicare/Medicaid healthcare plan with MHD needs”	*n* = 130	3.78 (5.52)	1.77 (0.88)	25.36 (31.34)
*F*, *p*-value		8.55, < 0.001	22.72, < 0.001	10.76, < 0.001

Note. Mean (SD) presented

**Table 3:** Service utilization differences by cluster

Clusters		Number of Psychotherapy Sessions	Number of Psychiatric Eval & Tx Sessions	Number of Group Therapy Sessions
Cluster 1: “Medicare/Medicaid healthcare plan with SUD needs”	*n* = 82	• < cluster 3 (*p* < 0.001) • < cluster 5 (*p* < 0.001)	• < cluster 2 (*p* < 0.001) • < cluster 3 (*p* < 0.001)	• < cluster 3 (*p* < 0.001)
Cluster 2: “Private pay and Charity Care healthcare plan with cocaine use disorder needs”	*n* = 117	• < cluster 3 (*p* = 0.023) • < cluster 5 (*p* = 0.022)	Significantly higher utilization than all other clusters except clusters 3 and 4. • > cluster 1 (*p* < 0.001) • > cluster 5 (*p* < 0.001) • > cluster 6 (*p* = 0.007)	• < cluster 3 (*p* = 0.002)
Cluster 3: “Medicare/Medicaid and other publicly-funded healthcare plans with mood disorder needs”	*n* = 120	Significantly higher utilization than all other clusters except cluster 5. • > cluster 1 (*p* < 0.001) • > cluster 2 (*p* = 0.023) • > cluster 4 (*p* = 0.008) • > cluster 6 (*p* < 0.001)	Significantly higher utilization than all other clusters except cluster 2. • > cluster 1 (*p* < 0.001) • > cluster 4 (*p* < 0.001) • > cluster 5 (*p* < 0.001) • > cluster 6 (*p* < 0.001)	Significantly higher utilization than all other clusters. • > cluster 1 (*p* < 0.001) • > cluster 2 (*p* = 0.002) • > cluster 4 (*p* < 0.001) • > cluster 5 (*p* < 0.001) • > cluster 6 (*p* < 0.001)
Cluster 4: “Private insurance + with low co-occurring needs”	*n* = 121	• < cluster 3 (*p* = 0.008) • < cluster 5 (*p* = 0.008)	Significantly higher utilization than cluster 5. • < cluster 3 (*p* < 0.001) • > cluster 5 (*p* < 0.001)	• < cluster 3 (*p* < 0.001)
Cluster 5: “Other publicly-funded healthcare plan with cannabis use disorder needs”	*n* = 135	Significantly higher utilization than all other clusters except cluster 3. • > cluster 1 (*p* < 0.001) • > cluster 2 (*p* = 0.022) • > cluster 4 (*p* = 0.008) • > cluster 6 (*p* < 0.001)	• < cluster 2 (*p* < 0.001) • < cluster 3 (*p* < 0.001) • < cluster 4 (*p* < 0.001) • < cluster 6 (*p* = 0.002)	• < cluster 3 (*p* < 0.001)
Cluster 6: “Medicare/Medicaid healthcare plan with MHD needs”	*n* = 130	• < cluster 3 (*p* < 0.001) • < cluster 5 (*p* < 0.001)	Significantly higher utilization than cluster 5. • < cluster 2 (*p* = 0.007) • < cluster 3 (*p* < 0.001) • > cluster 5 (*p* = 0.002)	• < cluster 3 (*p* < 0.001)

*Note. P* values reported from Bonferroni post hoc mean differences

## Discussion

This study examined a sample of adults with OUD receiving treatment from a community health center in a large urban area of NJ for a period of seven years after Medicaid expansion in this state. This study [1] characterized the sample [2], examined subtypes of the sample, and [3] identified differences in behavioral health services using CPT codes by subtype. Sample characteristics included high rates of co-diagnosed MHD and SUD across the analytic period. Approximately 72% of this study’s sample had a co-occurring SUD, and 39% had a co-occurring MHD. While this study included data across seven years, these percentages are comparable for co-occurring SUD and lower for co-occurring MHD than national 12-month estimates, which identified 77% co-occurring SUD and 64% co-occurring MHD among individuals with OUD [13]. However, the national estimates from another study identified that only approximately one-third of these individuals received treatment services in the past year [13], which is essential, considering our study includes adults with OUD receiving treatment.

Alongside the high prevalence of co-occurring MHD and SUD in the total sample, six specific groups were identified based on co-occurring diagnoses and how individuals paid for treatment. This study highlights the importance of regular assessments throughout the entire duration of treatment since clinical needs (e.g. diagnoses) and healthcare plans (e.g. Medicaid) may shift throughout an individual’s clinical journey. Further, identifying specific groupings based on medical records provides insight into where training and/or services should be expanded to emphasize: [1] dual diagnosis; [2] use of case management services to enroll persons onto publicly-funded healthcare plans over and above Medicare/Medicaid healthcare plans. Differing service utilization over the seven-year period was identified between the six different clusters further pointing to clinical need and access. Findings on service utilization differences by subtype demonstrate that the patient’s healthcare plan influenced the service types and number of sessions received. That influence played a more pivotal role than the patient’s mental health, substance use, or comorbidity needs. It is therefore of high importance to consider the needs of different individuals and how subtypes based on co-occurring diagnoses and healthcare plans inform clinical services provided. In particular, the most common forms of “other” public funding were mandated options (i.e. court-ordered). Because individuals receiving “other” public funding utilized more services than those on Medicare/Medicaid-specific public funding alone, it is important that providers and clinic administrators emphasize treatment engagement for those who are publicly funded but not receiving resources through mandated programs. This may include clinicians completing a chart review of payment type prior to providing care and incorporating strategies such as [1] motivational assessments to gauge treatment engagement; [2] education about available resources (e.g. other therapeutic services offered at the facility; public funding for transportation to/from the clinic) for those funded through Medicare/Medicaid alone.

### Limitations

These results may not be generalizable to all individuals with OUD receiving treatment. First, this subsample was required to be screened for substance misuse, which may omit some people with SUDs. Second, rates of MHDs such as depressive disorder may be underestimated because of less stringent and/or uniform assessment protocols for non-SUD diagnoses. Third, we grouped MHDs and SUDs together due to poor clustering, which may limit the ability to identify information about the role of specific diagnoses on cluster differences. Another limitation of this study is that it utilizes retrospective administrative data from a community health center. As such, the data are primarily collected for clinical and reimbursement purposes and not for research. Therefore, the researchers are not able to include a priori validated measures that examine factors such as craving, years of substance use, or other relevant factors that differentiate clusters. Chart review data at SUD specialty clinics has similar limitations, though they often have some variables regarding substance use history from non-validated scales [32]. By comparison, chart review data from a community mental health center in this context only included demographics, diagnoses, and healthcare plan information. As a result this analysis only includes diagnostic and healthcare data in the cluster solution. Although studies have pointed to limitations of using administrative data [33–35], data reflecting real-world clinical diagnoses may also be seen as a strength. Although diagnoses were examined, no data regarding severity specifiers were examined, nor were specific types of medications received for OUD made available in the dataset by the clinical partner. This is, however, how national-level real-world treatment data are often presented, even federal-level data provided by the Substance Abuse and Mental Health Services Administration [26]. Another limitation is this study focused on conditions co-occurring during the seven-year analytic period and not within the same twelve-month period [1]. 

## Conclusions

Identifying specific groupings of persons with OUD may improve services at community health centers [36]. The co-occurring conditions identified in this sample highlight the importance of comprehensive and integrated treatment for MHD and SUDs, particularly for those in underresourced communities. Notably, healthcare coverage, a socioeconomic factor that impacts access to care, played a critical role in distinguishing treatment needs and utilization. The varied and overlapping healthcare plans used for treatment point to a reality of clinical care.

## Electronic supplementary material

Below is the link to the electronic supplementary material.

Supplementary Material 1

## Data Availability

To comply with research ethics, the data underlying this study will not be made available to persons outside of the research team approved by the Rutgers University Institutional Review Board. Please contact the Principal Investigator, Dr. Jamey Lister, for any data inquiries.
