# Supplementary material for: Subtypes and service utilization among opioid use disorder patients at a community health center: findings from a medically underserved urban area of the Northeastern United States
Source: Addict Sci Clin Pract. 2025 May 8;20:39. doi: 10.1186/s13722-025-00564-z (PMC12060499; doi:10.1186/s13722-025-00564-z)
Supplement: Supplementary file 1 — Supplementary Material 1 [file 13722_2025_564_MOESM1_ESM.docx]

| ICD-10 Code | Diagnosis | Diagnostic Category |
| --- | --- | --- |
| F902 | Attention-deficit hyperactivity disorder, combined type | Attention-Deficit Hyperactivity disorders |
| F908 | Attention-deficit hyperactivity disorder, other type | Attention-Deficit Hyperactivity disorders |
| F901 | Attention-deficit hyperactivity disorder, predominantly hyperactive type | Attention-Deficit Hyperactivity disorders |
| F900 | Attention-deficit hyperactivity disorder, predominantly inattentive type | Attention-Deficit Hyperactivity disorders |
| F909 | Attention-deficit hyperactivity disorder, unspecified type | Attention-Deficit Hyperactivity disorders |
| F10120 | Alcohol abuse with intoxication, uncomplicated | Alcohol Use disorders |
| F1011 | Alcohol abuse, in remission | Alcohol Use disorders |
| F1010 | Alcohol abuse, uncomplicated | Alcohol Use disorders |
| F10280 | Alcohol dependence with alcohol-induced anxiety disorder | Alcohol Use disorders |
| F1024 | Alcohol dependence with alcohol-induced mood disorder | Alcohol Use disorders |
| F1021 | Alcohol dependence, in remission | Alcohol Use disorders |
| F1020 | Alcohol dependence, uncomplicated | Alcohol Use disorders |
| F1094 | Alcohol use, unspecified with alcohol-induced mood disorder | Alcohol Use disorders |
| F1099 | Alcohol use, unspecified with unspecified alcohol-induced disorder | Alcohol Use disorders |
| F064 | Anxiety disorder due to known physiological condition | Anxiety disorders |
| F419 | Anxiety disorder, unspecified | Anxiety disorders |
| F4000 | Agoraphobia, unspecified | Anxiety disorders |
| F411 | Generalized anxiety disorder | Anxiety disorders |
| F410 | Panic disorder [episodic paroxysmal anxiety] | Anxiety disorders |
| F410 | Panic disorder [episodic paroxysmal anxiety] without agoraphobia | Anxiety disorders |
| F418 | Other specified anxiety disorders | Anxiety disorders |
| F4010 | Social phobia, unspecified | Anxiety disorders |
| F3130 | Bipolar disorder, current episode depressed, mild or moderate severity, unspecified | Bipolar disorders |
| F3132 | Bipolar disorder, current episode depressed, moderate | Bipolar disorders |
| F315 | Bipolar disorder, current episode depressed, severe, with psychotic features | Bipolar disorders |
| F314 | Bipolar disorder, current episode depressed, severe, without psychotic features | Bipolar disorders |
| F310 | Bipolar disorder, current episode hypomanic | Bipolar disorders |
| F312 | Bipolar disorder, current episode manic severe with psychotic features | Bipolar disorders |
| F3111 | Bipolar disorder, current episode manic without psychotic features, mild | Bipolar disorders |
| F3112 | Bipolar disorder, current episode manic without psychotic features, moderate | Bipolar disorders |
| F3113 | Bipolar disorder, current episode manic without psychotic features, severe | Bipolar disorders |
| F3176 | Bipolar disorder, in full remission, most recent episode depressed | Bipolar disorders |
| F3174 | Bipolar disorder, in full remission, most recent episode manic | Bipolar disorders |
| F3175 | Bipolar disorder, in partial remission, most recent episode depressed | Bipolar disorders |
| F319 | Bipolar disorder, unspecified | Bipolar disorders |
| F3181 | Bipolar II disorder | Bipolar disorders |
| F3189 | Other bipolar disorder | Bipolar disorders |
| F1211 | Cannabis abuse, in remission | Cannabis Use disorders |
| F1210 | Cannabis abuse, uncomplicated | Cannabis Use disorders |
| F12259 | Cannabis dependence with psychotic disorder, unspecified | Cannabis Use disorders |
| F1221 | Cannabis dependence, in remission | Cannabis Use disorders |
| F1220 | Cannabis dependence, uncomplicated | Cannabis Use disorders |
| F12950 | Cannabis use, unspecified with psychotic disorder with delusions | Cannabis Use disorders |
| F1299 | Cannabis use, unspecified with unspecified cannabis-induced disorder | Cannabis Use disorders |
| F1419 | Cocaine abuse with unspecified cocaine-induced disorder | Cannabis Use disorders |
| F1411 | Cocaine abuse, in remission | Cannabis Use disorders |
| F1410 | Cocaine abuse, uncomplicated | Cannabis Use disorders |
| F1429 | Cocaine dependence with unspecified cocaine-induced disorder | Cannabis Use disorders |
| F1421 | Cocaine dependence, in remission | Cannabis Use disorders |
| F1420 | Cocaine dependence, uncomplicated | Cannabis Use disorders |
| F1490 | Cocaine use, unspecified, uncomplicated | Cannabis Use disorders |
| F332 | Major depressive disorder, recurrent severe without psychotic features | Depressive disorders |
| F3341 | Major depressive disorder, recurrent, in partial remission | Depressive disorders |
| F330 | Major depressive disorder, recurrent, mild | Depressive disorders |
| F331 | Major depressive disorder, recurrent, moderate | Depressive disorders |
| F333 | Major depressive disorder, recurrent, severe with psychotic symptoms | Depressive disorders |
| F339 | Major depressive disorder, recurrent, unspecified | Depressive disorders |
| F320 | Major depressive disorder, single episode, mild | Depressive disorders |
| F321 | Major depressive disorder, single episode, moderate | Depressive disorders |
| F323 | Major depressive disorder, single episode, severe with psychotic features | Depressive disorders |
| F322 | Major depressive disorder, single episode, severe without psychotic features | Depressive disorders |
| F329 | Major depressive disorder, single episode, unspecified | Depressive disorders |
| F3481 | Disruptive mood dysregulation disorder | Depressive disorders |
| F341 | Dysthymic disorder | Depressive disorders |
| F328 | Other depressive episodes | Depressive disorders |
| F3289 | Other specified depressive episodes | Depressive disorders |
| F919 | Conduct disorder, unspecified | Disruptive, Impulse-Control, and Conduct disorders |
| F639 | Impulse disorder, unspecified | Disruptive, Impulse-Control, and Conduct disorders |
| F6381 | Intermittent explosive disorder | Disruptive, Impulse-Control, and Conduct disorders |
| F632 | Kleptomania | Disruptive, Impulse-Control, and Conduct disorders |
| F913 | Oppositional defiant disorder | Disruptive, Impulse-Control, and Conduct disorders |
| F918 | Other conduct disorders | Disruptive, Impulse-Control, and Conduct disorders |
| F509 | Eating disorder, unspecified | Eating disorders |
| F630 | Pathological gambling | Gambling disorders |
| F1611 | Hallucinogen abuse, in remission | Hallucinogen Use disorders |
| F1610 | Hallucinogen abuse, uncomplicated | Hallucinogen Use disorders |
| F1621 | Hallucinogen dependence, in remission | Hallucinogen Use disorders |
| F1620 | Hallucinogen dependence, uncomplicated | Hallucinogen Use disorders |
| F1699 | Hallucinogen use, unspecified with unspecified hallucinogen-induced disorder | Hallucinogen Use disorders |
| F1690 | Hallucinogen use, unspecified, uncomplicated | Hallucinogen Use disorders |
| F1820 | Inhalant dependence, uncomplicated | Inhalant Use disorders |
| F952 | Tourette's disorder | Motor disorders |
| F8081 | Childhood onset fluency disorder | Neurodevelopmental disorders |
| Z134 | Encntr screen for certain developmental disorders in chldhd | Neurodevelopmental disorders |
| F8181 | Disorder of written expression | Neurodevelopmental disorders |
| F810 | Specific reading disorder | Neurodevelopmental disorders |
| F17200 | Nicotine dependence, unspecified, uncomplicated | Nicotine Use disorders |
| F17209 | Nicotine dependence, unspecified, with unspecified nicotine-induced disorders | Nicotine Use disorders |
| F42 | Obsessive-compulsive disorder | Obsessive-Compulsive and Related disorders |
| F429 | Obsessive-compulsive disorder, unspecified | Obsessive-Compulsive and Related disorders |
| F633 | Trichotillomania | Obsessive-Compulsive and Related disorders |
| F422 | Mixed obsessional thoughts and acts | Obsessive-Compulsive and Related disorders |
| F1111 | Opioid abuse, in remission | Opioid Use disorders |
| F1110 | Opioid abuse, uncomplicated | Opioid Use disorders |
| F1124 | Opioid dependence with opioid-induced mood disorder | Opioid Use disorders |
| F1121 | Opioid dependence, in remission | Opioid Use disorders |
| F1120 | Opioid dependence, uncomplicated | Opioid Use disorders |
| F11982 | Opioid use, unspecified with opioid-induced sleep disorder | Opioid Use disorders |
| F1199 | Opioid use, unspecified with unspecified opioid-induced disorder | Opioid Use disorders |
| F1599 | Oth stimulant use, unsp with unsp stimulant-induced disorder | Other Stimulant Use disorders |
| F1511 | Other stimulant abuse, in remission | Other Stimulant Use disorders |
| F1510 | Other stimulant abuse, uncomplicated | Other Stimulant Use disorders |
| F1521 | Other stimulant dependence, in remission | Other Stimulant Use disorders |
| F1520 | Other stimulant dependence, uncomplicated | Other Stimulant Use disorders |
| F1599 | Other stimulant use, unspecified with unspecified stimulant-induced disorder | Other Stimulant Use disorders |
| F1590 | Other stimulant use, unspecified, uncomplicated | Other Stimulant Use disorders |
| F1910 | Other psychoactive substance abuse, uncomplicated | Other Substance Use disorders |
| F1921 | Other psychoactive substance dependence, in remission | Other Substance Use disorders |
| F1920 | Other psychoactive substance dependence, uncomplicated | Other Substance Use disorders |
| F1994 | Other psychoactive substance use, unspecified with psychoactive substance-induced mood disorder | Other Substance Use disorders |
| F19950 | Other psychoactive substance use, unspecified with psychoactive substance-induced psychotic disorder with delusions | Other Substance Use disorders |
| F19959 | Other psychoactive substance use, unspecified with psychoactive substance-induced psychotic disorder, unspecified | Other Substance Use disorders |
| F1999 | Other psychoactive substance use, unspecified with unspecified psychoactive substance-induced disorder | Other Substance Use disorders |
| F1994 | Oth psychoactive substance use, unsp w mood disorder | Other Substance Use disorders |
| F19959 | Oth psychoactv substance use, unsp w psych disorder, unsp | Other Substance Use disorders |
| F603 | Borderline personality disorder | Personality disorders |
| F602 | Antisocial personality disorder | Personality disorders |
| F6089 | Other specific personality disorders | Personality disorders |
| F250 | Schizoaffective disorder, bipolar type | Schizophrenia Spectrum |
| F251 | Schizoaffective disorder, depressive type | Schizophrenia Spectrum |
| F259 | Schizoaffective disorder, unspecified | Schizophrenia Spectrum |
| F209 | Schizophrenia, unspecified | Schizophrenia Spectrum |
| F2081 | Schizophreniform disorder | Schizophrenia Spectrum |
| F1311 | Sedative, hypnotic or anxiolytic abuse, in remission | Sedative Use disorders |
| F1310 | Sedative, hypnotic or anxiolytic abuse, uncomplicated | Sedative Use disorders |
| F1321 | Sedative, hypnotic or anxiolytic dependence, in remission | Sedative Use disorders |
| F1320 | Sedative, hypnotic or anxiolytic dependence, uncomplicated | Sedative Use disorders |
| F1399 | Sedative, hypnotic or anxiolytic use, unspecified with unspecified sedative, hypnotic or anxiolytic-induced disorder | Sedative Use disorders |
| F1390 | Sedative, hypnotic, or anxiolytic use, unspecified, uncomplicated | Sedative Use disorders |
| F5101 | Primary insomnia | Sleep Wake disorders |
| F4321 | Adjustment disorder with depressed mood | Trauma and stressor Related disorders |
| F4323 | Adjustment disorder with mixed anxiety and depressed mood | Trauma and stressor Related disorders |
| F4329 | Adjustment disorder with other symptoms | Trauma and stressor Related disorders |
| F4320 | Adjustment disorder, unspecified | Trauma and stressor Related disorders |
| F4310 | Post-traumatic stress disorder, unspecified | Trauma and stressor Related disorders |
| F439 | Reaction to severe stress, unspecified | Trauma and stressor Related disorders |
| F09 | Unspecified mental disorder due to known physiological condition | Unspecified Psychiatric disorders |
| F39 | Unspecified mood [affective] disorder | Unspecified Psychiatric disorders |
| F99 | Mental disorder, not otherwise specified | Unspecified Psychiatric disorders |
| F348 | Other persistent mood [affective] disorders | Unspecified Psychiatric disorders |
| Z8659 | Personal history of other mental and behavioral disorders | Unspecified Psychiatric disorders |
| F54 | Psychological and behavioral factors associated with disorders or diseases classified elsewhere | Unspecified Psychiatric disorders |
